# Statistical Learning as a Tool for Rehabilitation in Spatial Neglect

**DOI:** 10.3389/fnhum.2013.00224

**Published:** 2013-05-29

**Authors:** Albulena Shaqiri, Britt Anderson, James Danckert

**Affiliations:** ^1^Department of Psychology, Cognitive Neuroscience, University of Waterloo, Waterloo, ON, Canada; ^2^Center for Theoretical Neuroscience, University of Waterloo, Waterloo, ON, Canada

**Keywords:** spatial neglect, rehabilitation, priming, statistical learning, updating

## Abstract

We propose that neglect includes a disorder of representational updating. Representational updating refers to our ability to build mental models and adapt those models to changing experience. This updating ability depends on the processes of priming, working memory, and statistical learning. These processes in turn interact with our capabilities for sustained attention and precise temporal processing. We review evidence showing that all these non-spatial abilities are impaired in neglect, and we discuss how recognition of such deficits can lead to novel approaches for rehabilitating neglect.

## Introduction

The spatial impairments of neglect are striking and have dominated most research until the past few years (Pisella and Mattingley, 2004; Corbetta et al., 2005; Danckert and Ferber, 2006; Karnath and Rorden, 2012). As a result, a large number of rehabilitation programs, such as prism adaptation and vestibular stimulation, have focused on correcting those deficits (Luauté et al., 2006; Redding and Wallace, 2006; Bowen et al., 2007; Kerkhoff and Schenk, 2012). Unfortunately, success has been limited. This suggests that non-spatial impairments in neglect may contribute to its rehabilitory recalcitrance. Based on the results of recent studies, we have hypothesized that one such non-spatial deficit in neglect is the meta-level impairment of mental model building and updating, also referred to as representational updating (Danckert et al., 2012b).

Our everyday life is guided by regularities in the environment and also our ability to notice and adapt to those regularities: we dress with warm clothes if it has been snowing; based on our previous experiences, we guess what the weather will be like for the next few days. But if we have to visit a warm country, then we adapt to the new context and build a new model of the weather and the clothes needed for the higher temperatures.

The ability to learn environmental regularities and to be sensitive to their relationships is essential for building mental models. Detecting when a context has changed is the signal that a mental model needs to be adapted to the new context and updated. Therefore, representational updating impairments are revealed by the inability to learn environmental statistics. Ultimately, an impairment in this process leads to incorrect interactions with the environment, poor predictions about future states of the world, and an impaired ability to benefit from instruction and experience.

The ability to build successful representations depends on a number of interdependent sub-processes, where one of the most important is statistical learning: the ability to learn that some elements occur more often than others. Statistical learning in turn requires other, more elemental processes, such as priming. In addition, priming and statistical learning rely on intact temporal processing and working memory: to detect regularities in our environment, as for example whether something is frequently repeating its position, we must remember what has happened and be accurate in judging if it has occurred recently.

Working memory or temporal processing deficits, as well as difficulties in position priming and statistical learning, can all lead to a representational updating deficit. Those processes have also been demonstrated to be impaired in spatial neglect, which points the way to new tactics and targets that can be the focus of rehabilitation for this disorder.

In our review, we accept as givens that neglect is phenomenally heterogeneous, and that spatial impairments form the definitional core for the disorder. As the spatial components of neglect are well reviewed elsewhere (e.g., Danckert and Ferber, 2006; Karnath and Rorden, 2012), we do not review them here. Therefore, our review focuses on studies demonstrating non-spatial deficits in neglect. We show that those deficits in neglect include impaired priming, temporal processing, visual and auditory statistical learning, and working memory. We interpret other non-spatial impairments of neglect, such as prolonged attentional blinks and decreased sustained attention, as reflecting similar impairments. Lastly, we review evidence for updating impairments in neglect and conclude by suggesting that the hypothesis of neglect as a disorder of representational updating highlights new approaches for rehabilitation.

## Non-Lateralized Deficits in Spatial Neglect

Numerous recent studies have demonstrated deficits in neglect that are not lateralized spatially, but that contribute to the complexity of this disorder (Husain et al., 1997; Becchio and Bertone, 2006; Malhotra et al., 2006; Ptak et al., 2007). Husain and Rorden (2003) suggest that a combination of non-lateralized and lateralized deficits might explain the difficulty in finding effective rehabilitation strategies.

The interest in non-lateralized deficits in neglect has grown in recent years with studies demonstrating a number of fundamental non-spatial impairments, such as decreased arousal, problems with sustained attention, spatial working memory impairments, and non-spatial attentional biases (for a review, see Husain and Rorden, 2003; Corbetta and Shulman, 2011; Danckert et al., 2012b).

Many neglect patients show decreased arousal and vigilance; this translates into a lower level of sustained attention (Robertson et al., 1997; Farné et al., 2004; Corbetta and Shulman, 2011). Several studies have implicated the right hemisphere in deficits of arousal or alertness (Robertson et al., 1995, 1997; Rueckert and Grafman, 1998; Sturm and Willmes, 2001; Corbetta et al., 2005; Fimm et al., 2006; Grahn and Manly, 2012). A correlation between neglect and a decreased level of sustained attention was first shown by Heilman and Valenstein (1979) and has been confirmed by multiple studies (Hjaltason et al., 1996; Robertson et al., 1997; Samuelsson et al., 1998; Barrett et al., 2007). In a study where right brain damaged patients were asked to count a series of tones, Robertson et al. (1997) found a correlation between sustained attention and the bias in spatial attention, confirming a connection between spatial and non-spatial aspects of neglect.

Another non-lateralized deficit that could contribute to spatial biases in neglect is a deficit of spatial working memory. Husain et al. (2001) recorded a neglect patient’s eye movements while the patient judged whether a stimulus had been seen before. The authors found that a patient suffering from left neglect revisited old targets and identified them as new, even when they were presented on the right, ipsilesional side.

Neglect patients were also impaired when tested in vertical spatial working memory tasks, even though there was no left-right spatial component (Ferber and Danckert, 2006; Malhotra et al., 2006). The working memory deficit predicted the general degree of impairment in patients with neglect: the less patients can retain of their previous actions, the less liable are they are to undertake new actions (Husain et al., 2001).

An additional non-spatial impairment in neglect that has been linked to working memory is a prolonged attentional blink (Johnston et al., 2012). The attentional blink refers to the observation that when a person must detect multiple targets, the correct detection of one target impairs the ability to detect a subsequent target that follows it shortly thereafter in time. A recovery interval of between 200 and 500 ms is necessary for the detection of a second target to return to baseline (Dux and Marois, 2009). For many neglect patients, this interval is two to three times longer: after they have detected the first target, neglect patients are not aware of the second target unless there is an interval of about 1200 ms (Raymond et al., 1992; Husain et al., 1997; Shapiro et al., 1997, 2002; Johnston et al., 2012).

The non-lateralized deficits just highlighted demonstrate that neglect is more than a spatial disorder. We suggest that many of these different symptoms are related and reflect a mutual dependence. We now review data demonstrating that neglect patients also have impairments in priming, temporal processing, statistical learning and working memory, and suggest that those different deficits sum to a representational updating impairment.

## Neglect as a Disorder of Generating and Updating Mental Models

### Position priming

Studies in visual search are greatly influenced by the research in priming and how the effect of the repetition of the target position or features influence participants’ reaction time (for a review, see Neely, 1991; Kristjansson, 2008; Kristjánsson and Campana, 2010). Maljkovic and Nakayama (1994, 1996) were the first to report that when the features or the position of a target are repeated, participants are faster to detect it. In their study of position priming (1996), participants searched for a diamond with its left or right corner missing. There were two distractors. The stimuli were placed in an elliptical organization, and the target either repeated or switched its position on successive trials. The authors found that when the target was presented in the same position on successive trials, participants were faster and more accurate to respond than when the target’s location was switched. Other studies have since confirmed those results for the priming of context, object features, movement, and presentation interval (Chun and Jiang, 1998; Goolsby and Suzuki, 2001; Los and Van Den Heuvel, 2001). The priming effect has also been tested in neglect patients (Kristjánsson et al., 2005; Saevarsson et al., 2008; Shaqiri and Anderson, 2012a,b). Saevarsson et al. (2008) repeated or switched the overall context in which a target was presented. They found a preserved priming effect in neglect. In their second experiment, they tested the priming effect in contralesional and ipsilesional space by repeating the context in both visual fields. Patients were faster to detect targets when the context was repeated, even when the presentation was in contralesional space.

**Figure 1:**
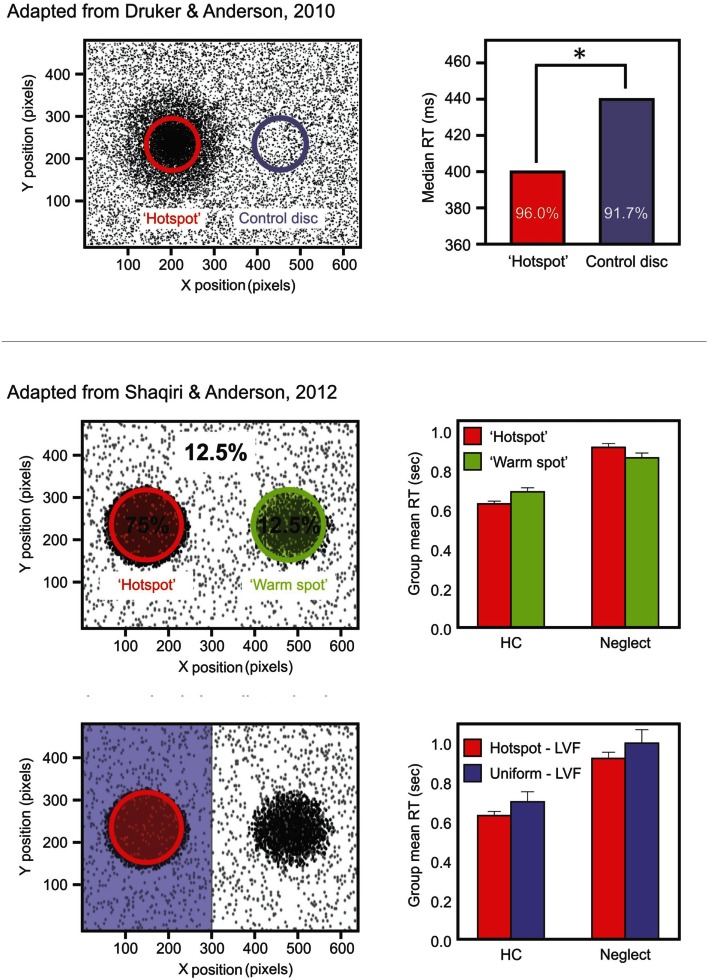
**Upper panels show data from the study of Druker and Anderson (2010) testing undergraduate students**. Participants made color discriminations for targets that could appear anywhere on the screen but were more likely to come from a high probability “hotspot” region. Results (right panel) showed faster RTs and increased accuracy for targets in the hotspot despite participants being unaware of this high probability region. Middle panels show data from a different study: Shaqiri and Anderson (2012a). This is a modified version of the Druker and Anderson (2010) task in a group of healthy older controls (HCs) and right brain damaged (RBD) patients with Neglect. Contrary to HCs, Neglect patients failed to show a benefit in RT for targets presented in a contralesional, high probability region (Shaqiri and Anderson, 2012a). Lower panels show HCs and neglect patients’ RT for the hotspot and the rest of the left-sided trials: although overall slower on the left, Neglect patients were sensitive to the biased distribution of the target.

We recently tested color and position priming in neglect with patients discriminating the color of a dot that could be either black or white (Shaqiri and Anderson, 2012a). Stimuli were biased to appear 75% of the time in a high probability region on the left side of space. Our results demonstrated that neglect patients had a preserved color priming, but their results for location priming were less consistent. Indeed, when the target repeated the same position, participants did not show significantly faster RTs than when the target was presented in another location – although there was a trend – which demonstrated that the benefit from position priming in neglect patients was attenuated (Figure 1). This was not the case for color priming: when the target repeated the same color, participants were faster to respond, even if the target appeared in contralesional space.

Although we did not control for eye position, we believe that the difficulty of neglect patients to benefit from position priming is not due to a remapping impairment, as has been hypothesized by Pisella and Mattingley (2004). In their review paper, the authors suggest that patients’ gaze-shifts toward their contralesional side degrade all previously visited and remembered locations, creating a remapping problem. This hypothesis has been contradicted by the study of Vuilleumier et al. (2007), who tested how gaze-shifts affect the memory of location in neglect patients. The study revealed results that were different from the hypothesis of Pisella and Mattingley (2004), as Vuilleumier et al. (2007) found that only gaze-shifts to the far right affect the location information in neglect patients, but when patients had to make a left gaze-shift, they showed a preserved ability to maintain and update the location information (see also Vasquez and Danckert, 2008 for similar results in healthy individuals). The results of Vuilleumier et al. (2007) consolidate our results of position priming. Indeed, we presented 75% of the targets on the patients’ contralesional side (Shaqiri and Anderson, 2012a), therefore, we believe that patients’ difficulty to benefit from position priming is not a demonstration of their remapping impairment, but is a more generic impairment of updating and benefiting from regularities of the environment.

These results are in accordance with Kristjánsson et al. (2005), who had neglect patients detect a distinctly colored diamond and report whether the top or the bottom corner was missing. The three diamonds were presented in a triangular array (i.e., bottom left, bottom right, and top middle). The authors found preserved color and position priming when participants had an unlimited time to respond to the target, although one of the two patients needed at least three repeats of the same position to show a priming effect. Moreover, when the time of the display was limited to 200 ms, patients did not show a position priming effect, unless they indicated that they had consciously detected the target, whereas color priming remained intact regardless of stimulus duration. Kristjánsson et al. (2005) concluded that awareness was necessary for patients to show position priming on their left side.

**Figure 2:**
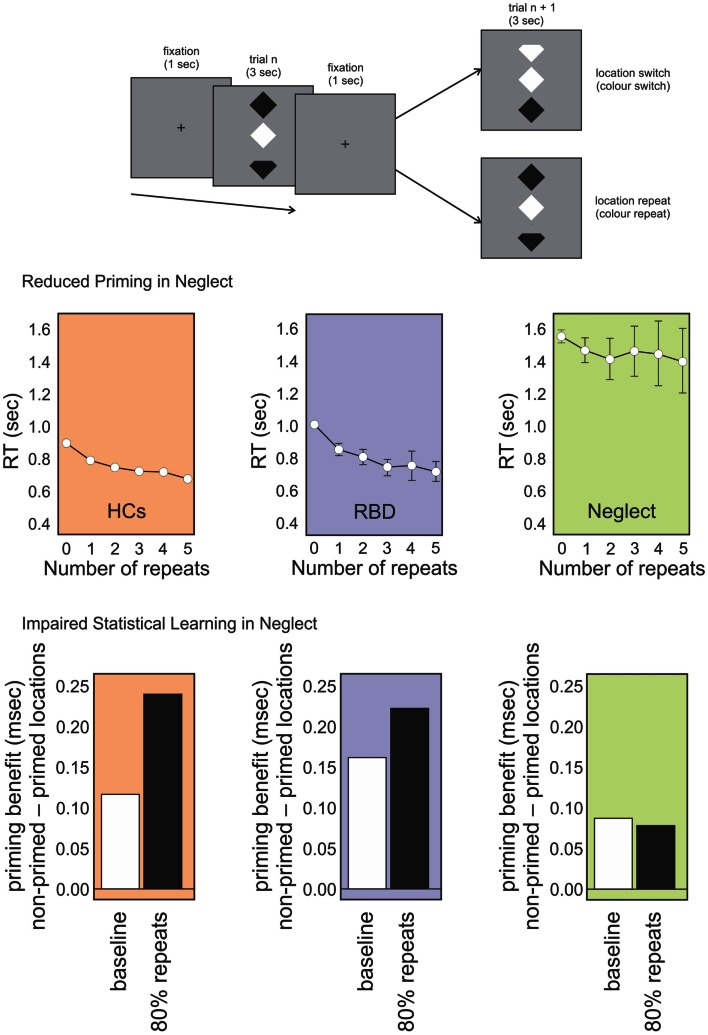
**Topmost panel: schematic representation of our position priming task (Shaqiri and Anderson under review)**. Participants were required to detect if the top or bottom notch of the odd-colored diamond was missing (the schematic here exaggerates the actual physical distinction). Middle panel: RT data for healthy controls (HCs; orange panel), RBD patients (purple panel), and Neglect patients (green panel) for targets that repeated spatial locations on subsequent trials (up to five repeats). RBD patients show reduced priming relative to HCs who show increased priming over all five trials. In contrast, Neglect patients show no priming benefit after trial 1. Lower panel: priming benefit in conditions where repeated locations and switched locations are equally likely (baseline; white bars) vs. conditions in which location repeats were highly probably (i.e., location repeated on 80% of trials). Controls and RBD patients show an increased priming benefit on the highly probable repeat trials whereas Neglect patients do not.

These studies included a spatial aspect in their design, as they presented stimuli on the contralesional and ipsilesional side. This complicates the interpretation of the impairment. In order to avoid a spatial bias, we adapted Maljkovic and Nakayama’s (1996) study by presenting the target and distractors vertically aligned in central space (Figure 2). We assessed whether patients had preserved position priming, that is, if they were faster when the target repeated the same position successively (Shaqiri and Anderson, 2012b, under review). We found that although neglect patients had an overall priming effect, the magnitude was reduced compared to healthy controls. Further, the benefit did not show an increase with multiple spatial repeats, an effect that was seen with controls. Thus, a deficit in position priming was revealed in a task that eliminated lateral spatial biases (Figure 2). A generic priming deficit was not present though, as most studies, including our own, have demonstrated preserved color priming.

The brain regions associated with neglect may explain the differential results for color and position priming. In an fMRI study investigating the neural correlates of priming, Kristjánsson et al. (2007) found different brain regions activated by color and position priming conditions. While both of these priming effects were associated with regions traditionally linked with the control of attention, the so called “attention network” that includes the intraparietal sulci (Corbetta and Shulman, 2002, 2011), the color repetition condition also showed suppression of activity in the inferior temporal region. Position priming was more related with regions such as the right inferior parietal cortex and frontal areas. Kristjánsson et al. (2007) also found a greater involvement of the right hemisphere for position priming than for color priming. Although there is no single brain region where damage is both necessary and sufficient for causing spatial neglect, the right inferior parietal and the frontal lobe are frequently involved in the strokes that produce neglect (Corbetta and Shulman, 2002; Ricci et al., 2012). The correspondence between the regions involved in position priming and those involved with spatial neglect may explain why patients do not show as robust position priming effects as do controls and why different studies might find varying results.

### Temporal deficits in neglect

The results discussed above reveal that neglect patients have difficulties benefiting from successive repeats of the same position by the target, and therefore demonstrate attenuated position priming. We make the hypothesis that this difficulty might be explained by the temporal processing impairments demonstrated by neglect patients (Berberovic et al., 2004; Danckert et al., 2007; Merrifield et al., 2010). Patients tend to underestimate multisecond intervals: Danckert et al. (2007) tested neglect patients in a temporal estimation task. Arranged in a circular shape, eight open circles were filled in one after another, following a clockwise motion. A trial could last 5, 15, 30, and 60 s and patients were asked how long the clockwise motion lasted on each trial. The authors found that neglect patients underestimated all durations, showing an impairment for estimating the passage of time: even for trials that lasted 60 s, neglect patients reported that the clockwise motion was present for no longer than 10 s. Those deficits have also been found in the processing of auditory stimuli (Cusack et al., 2000; Merrifield et al., 2010).

The temporal processing impairment is intrinsically linked with priming, as the importance of timing in priming has been demonstrated by Maljkovic and Nakayama (2000), who tested the ability of participants to benefit from position priming with different inter-trial intervals. While a break of 30 s between two trials did not affect priming magnitude, a break of 90 s did, as it reset any possible benefit from target position repetition to its initial pace. The authors demonstrated that priming was cumulative and that at short intervals (from 1 to 30 s) priming occurs, but at longer intervals there is some degradation of the implicit memory of previous information regarding target position. The difficulty neglect patients have in benefiting from more than one repeat of position in a priming task might be accounted for by temporal and memory impairments. To restate, since neglect patients have slower response times for any task in general (Kaizer et al., 1988; Shaqiri and Anderson, 2012a), it means that they have to keep in mind the association between the trials for a longer period of time and therefore, they are submitted to fewer trials from which they can accumulate information, compared to the healthy controls. A problem in keeping the relationship between the trials in their implicit memory might prevent patients from extrapolating to a more general regularity about their environment.

The importance of timing in the priming effect and the demonstration of an impairment in temporal processing in neglect affects other processes as well, such as statistical learning. Indeed, as we will demonstrate in the subsequent sections, priming, and statistical learning are closely related, to the point that some authors (Walthew and Gilchrist, 2006) have questioned whether statistical learning is not simply a form of priming, or if the latter is a necessary step for statistical learning to occur (Jones and Kaschak, 2012). We review different studies that have investigated the relationship between priming and statistical learning and how they are involved in building and updating mental models.

### Statistical learning

Statistical learning is a form of implicit learning that occurs through mere exposure and observation and does not involve explicit feedback (Turk-Browne et al., 2008; Aslin and Newport, 2012). It has been demonstrated for both auditory and visual modalities. Bulf et al. (2011) found that newborn infants were able to extract the transitional probabilities of simple visual structures: they presented pairs of shapes to babies using a higher transition probability within pairs of shapes and a lower transition probability between the shapes in a pair (for example, a circle was followed by a square 100% of the time, but the square was followed by a triangle or a diamond with equal probability). Results showed that the infants demonstrated preferential looking toward novel sequences. Bulf et al. (2011) concluded that newborns have the ability to detect regularities from the environment and learn which elements are being repeated more often.

Many early studies on statistical learning focused on very young children. Fiser and Aslin (2002), tested 9-month-old babies in a more complicated paradigm of visual statistical learning. They presented four base pairs of shapes combined with four noise elements, so that each baby was presented with consecutive base pairs and a noise element during the task. The data revealed that babies showed a greater preference for base pairs over non-base pairs, and the authors suggested that the infants learned the co-occurrence of the shapes.

While the phenomenon of statistical learning is well established, its relationship to priming is complex. When a statistical distribution leads to frequent repeats there are also more primed trials. Walthew and Gilchrist (2006) suggested that claims of statistical learning of spatial probability distributions in neglect might be explained on this basis; rather than learning underlying distributions, faster responses in areas of high probability could merely reflect the influence of a greater number of primed trials in those regions.

To address this issue and investigate further the relationship between priming and statistical learning, we conducted a study where undergraduate participants discriminated the color of a small dot. The main manipulation of the study was the spatial location of the target: 80% of the time stimuli were presented within a high probability region on one side of the display (Druker and Anderson, 2010). Participants were faster and more accurate to respond to targets presented in the high probability region compared to the rest of the screen. Given that exact locations were rarely if ever repeated (Figure 1), it is difficult to explain this result as simply a consequence of position priming: because target locations were free to be anywhere on the screen, and because targets were small, the risk of repeating target position was almost non-existent. Furthermore, a questionnaire administered at the end of testing revealed that participants were not aware of the biased location for the target, demonstrating that the statistical learning of the high probability target zone was achieved implicitly.

Statistical learning has also been assessed in neglect patients. Geng and Behrmann (2002, 2006), had neglect patients detect the letters L and F that appeared among distractors (letters T and E). Targets were biased to appear 80% of the time on one side of the computer screen. The authors found that neglect patients were faster at detecting targets that appeared in the high probability region, even if this was in contralesional space. The results of Geng and Behrmann (2002, 2006), give promise for the use of statistical learning as a rehabilitation strategy for neglect patients. This technique does not need supervision or feedback. Patients’ observations lead to an implicit learning of the distribution of elements in their environment. This could facilitate the direction of attention and help to overcome the ipsilesional attentional bias.

From this perspective we conducted two studies (Shaqiri and Anderson, 2012a,b, under review) where we tested statistical learning in neglect. Our first study adapted the paradigm of Druker and Anderson (2010) and tested whether neglect patients could learn a spatial statistical distribution and use it as an attentional cue in a color discrimination paradigm (Figure 1; Shaqiri and Anderson, 2012a). We biased the targets to appear 75% of the time in a high probability region on the left side of space. As was the case in our previous study in healthy controls (Druker and Anderson, 2010), target locations varied throughout the screen eliminating any concerns about position priming. Where priming did occur it was of a lesser magnitude in neglect patients compared to controls. To explore statistical learning in the same paradigm we first excluded trials where the previous target location was within 5 °of visual angle. With all trials considered, neglect patients were slower to respond to targets in the high (i.e., 75%) probability region in left space when compared with a low probability (12.5%) region in the mirror symmetric location in right space (Figure 1). With the primed trials removed, and considering only targets appearing in left, neglected space, we found that neglect patients were indeed sensitive to the high probability region of the screen (Figure 1). That is, when we compared the trials in the hot spot (i.e., the high probability region) with the other left-sided trials, neglect patients were faster to respond for the hot spot, although their RTs were slower compared with RTs to right-sided targets. These data demonstrated that patients are somewhat sensitive to the statistical distribution of targets, but also that they have difficulties benefiting from these regularities to the same extent as healthy controls (Figure 1).

In order to investigate whether the spatial elements of the task were central to the results, we tested neglect patients in a visual search task where we presented targets vertically in the middle of the screen (Figure 2; Shaqiri and Anderson, 2012b, under review). As with our previous task, here we could look both at priming and statistical learning within the same task. Priming was examined on trials in which target locations or colors repeated. To examine statistical learning we biased the transitional probability of stimuli positions to include a high repeat condition (an 80% probability of repeating target location), or a switch condition in which targets changed location on 80% of trials. As with our previous task that explicitly manipulated target locations throughout the visual field, results for this study showed that, contrary to healthy controls, who were faster to respond to targets on the high repeat condition, neglect patients did not learn the statistical distribution of the targets, independently from the spatial position of stimuli. Right brain damaged patients without neglect performed much like controls suggesting that the failure to benefit from statistical regularities (i.e., no RT benefit in the high repeat condition) was unique to neglect. This, despite the fact that *primed* trials were faster. In other words, the magnitude of the position priming effect was the same whether repeated trials were very likely or very unlikely (Figure 2). This demonstrates the difficulty neglect patients have in making use of environmental statistics and also that this difficulty is not simply a consequence of left-right biases of attention.

All these different paradigms tested the visual modality, but if such an impairment is generic, it ought to be present for other sensory modalities, given that numerous studies have reported multimodal impairments in neglect, including auditory and tactile deficits (for a review, see Pavani et al., 2003; Jacobs et al., 2012). For example, Cusack et al. (2000) found that neglect patients show auditory impairments for temporal aspects of stimuli, mapping a visual bias to the auditory modality (Bisiach et al., 1984; Tanaka et al., 1999), and they demonstrate a greater uncertainty for the location of sounds compared to healthy controls (Pavani et al., 2002).

**Figure 3:**
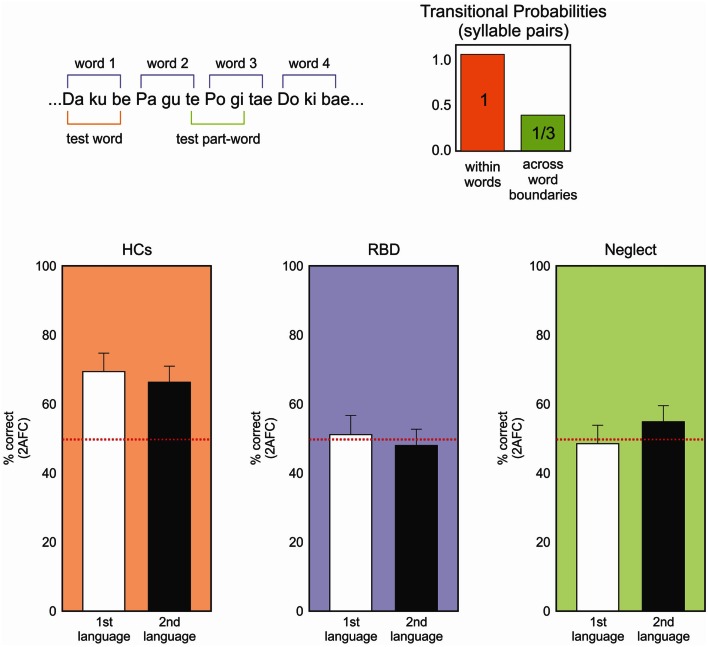
**Upper panel: representation of the nonsense language task**. Participants heard a constant stream of nonsense syllables (no temporal gaps between syllables) for ∼10 min. Afterward they make forced-choice discriminations of “words” and “non-words” constructed from the same syllables. Words are defined by transitional probabilities with syllable pairs within word boundaries having 100% association (ku always follows da) and between word boundaries having 33% probability across all other syllables. Lower panels: forced-choice discrimination performance for two nonsense languages. HCs (orange) clearly perform above chance (red dotted line) on both languages. Neither the RBD (purple) or neglect (green) patients can discriminate the languages – task that was well performed by 8-month-old infants (data from Anderson and Danckert, 2013; Shaqiri et al., in preparation).

Those studies, which demonstrated multimodal impairments in neglect, motivated our assessment of neglect patients’ ability to learn the transition probability of nonsense words in an auditory statistical learning paradigm (Figure 3; Anderson and Danckert, 2013; Shaqiri et al., in preparation). This procedure relied on decades of results on auditory statistical learning exemplified by Saffran et al. (1999) and Aslin et al. (1998). They exposed 8-month-old infants to tri-syllabic nonsense words (for example bidaku, padoti, golabu) where the transitional probability of syllables within words was 100% (e.g., “go” was always followed by “la,” “la” by “”bu” to create “golabu,” etc.). In contrast, the transitional probability for syllables between words was 33% (e.g., “bu” of “golabu” was followed by “pa,” “bi,” or “go” equally often). The words had no breaks between them and were presented by computer to avoid clues to the word borders other than the statistics of syllable transitions. The continuous stream of speech presented to the children lasted 2 min. Saffran et al. (1999) found that 8-month-old infants were able to identify the words, extracting information about the word boundaries solely on the basis of the transitional probability of those words.

This effect has been confirmed for adults. Gebhart et al. (2009) used a similar paradigm for university undergraduates; some participants heard two different languages (5 min each). Participants were exposed to either one language, both languages without a break, or both languages with a 30 s break between the first and second language. Undergraduates learned the first language, as they were able to correctly identify the words with 80% accuracy in a 16 item forced-choice test. When presented with two languages, they learned both as long as they had a break between the exposures to each.

Adapting the paradigm of Gebhart et al. (2009), we tested neglect patients for their ability to learn the transitional probability of the tri-syllabic nonsense words (Figure 3; Anderson and Danckert, 2013; Shaqiri et al., in preparation). For all the studies, neglect was assessed using the letter cancelation, line bisection, and figure coping from the Behavioral Inattention Tests (BIT) (Wilson et al., 1987). Patients were diagnosed as having neglect when they missed more than 10% of the letters on the left in the letter cancelation test, when the rightward bias was higher than 5% of the total length of the line and finally, when patients missed parts of the figures for the figure coping task. Based on those criteria, we recruited eight neglect patients (main age = 72, SD = 9.02): three had lesions of the parietal lobe, two with lesion of the temporo-parietal lobeu, and finally, three with fronto-parietal lesions. The neglect patients listened to the stream of nonsense words forming the two different languages. Four patients heard both languages without a break (10 min) and four listened to the two languages for 5 min each, with the two languages separated by a 30 s break. After listening to the language streams, participants were tested in a forced-choice format where the words they heard were paired with part words made-up of syllables that spanned word borders (Figure 3). Neglect patients did not show any learning effect. Indeed, patients did not perform the task above chance, contrary to our healthy controls who learned the transition probability between syllables and identified the correct words about 80% of the time. These results demonstrate that the difficulty neglect patients have in learning statistical distributions is multimodal and is neither limited to visual or spatially presented material. Our study did not involve spatial aspects, but tested the general ability of those patients to be sensitive to the transitional probability between the syllables within the word, an ability shown to be present in 8-month-old infants (Saffran et al., 1999).

### Working memory and statistical learning

The different studies we conducted on statistical learning (Shaqiri and Anderson, 2012a,b) confirmed that neglect patients have difficulties benefiting from statistical regularities. We hypothesized that this might be, in part, because of the temporal processing impairment demonstrated by those patients (see above), and in part from working memory impairments. Spatial working memory has been shown to be deficient in neglect patients (Husain et al., 1997; Ferber and Danckert, 2006; Johnston et al., 2012) but based on the different studies we conducted, we extend those findings and hypothesize that neglect patients might demonstrate working memory deficits that exceed the spatial scope and are more generic, which contributes to patients’ impairment of statistical learning.

**Figure 4:**
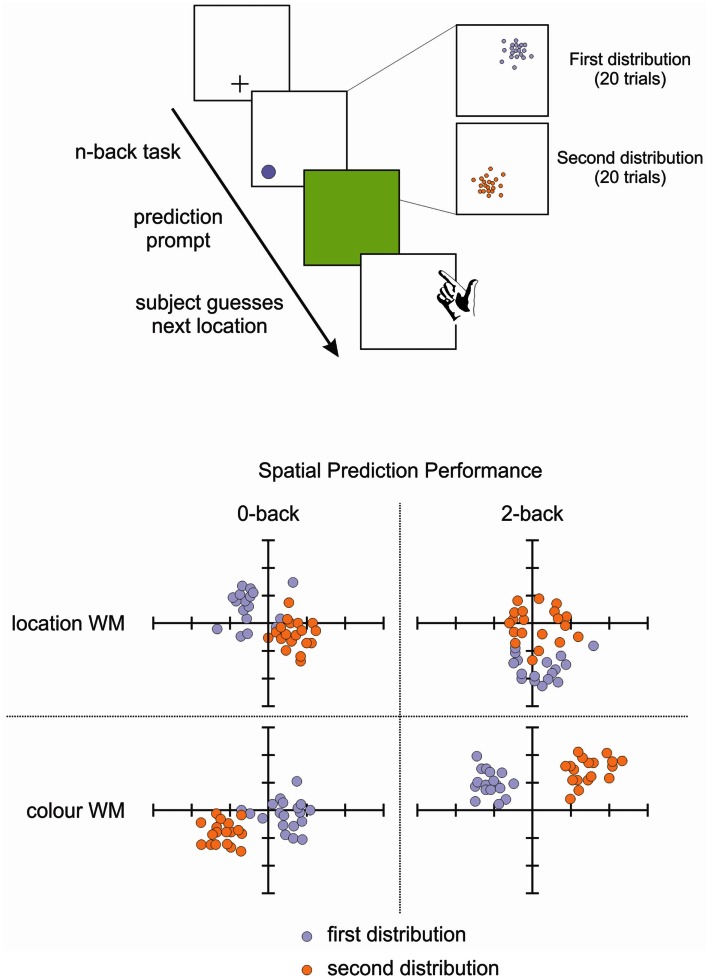
**Schematic representation of our spatial prediction and working memory task (Valadao et al., 2012)**. Participants first perform an n-back task related to the color or location of targets on a given trial. They are then required to predict the location of a target on the next trial. The distribution for target locations is chosen from 1 quadrant for 20 trials before being switched to another quadrant for 20 trials. Lower panel shows performance from a representative participant on the spatial prediction component of the task in the 0-back (left) and 2-back (right) tasks. The participant’s predictions were less accurate when performing the 2-back spatial working memory task (upper right panel).

To that end, we tested the involvement of working memory in statistical learning, in order to investigate whether these processes were interdependent and to what extent working memory plays a role in statistical learning. This study (Valadao et al., 2012) required participants to complete an n-back working memory task and a prediction task simultaneously. Participants had to predict the location of a target that was biased to appear in a specific quadrant of the display (Figure 4). They also had to do a 0-back or 2-back task based on the shape, location or color of the target, which tested feature and spatial working memory. We found that when participants did the 2-back task, they were not as accurate in learning the biased probability distribution of the target location, particularly if spatial working memory was involved. Another study that tested working memory while manipulating the statistical distribution of the target also found a close relationship between these two aspects: participants were better at storing in working memory targets that were presented within a high probability area, without necessarily being aware of this facilitation (Umemoto et al., 2010). These studies demonstrate that for statistical learning to occur, participants need free working memory resources. The impairment that neglect patients demonstrate in spatial working memory (Husain et al., 1997; Johnston et al., 2012) might extend and affect working memory more generally, which could contribute to the difficulty patients have in learning and benefiting from statistical regularities in their environment. If neglect patients cannot keep in memory the recent information about target locations and features, then they will not have access to the information necessary for building mental models of their environment. This difficulty in holding information in mind could also affect their ability to notice changes in the environment, changes that might require updating of mental models.

### Representational updating impairment in neglect

Combining the results of the various studies reviewed above, we hypothesize that neglect involves a disorder of representational updating (Danckert et al., 2012a,b) and consequently, that rehabilitation strategies need to address this deficit. Patients need to be trained to improve their ability to detect and exploit regularities within their environment. To interact efficiently with the environment, a representation of recent perceptual information is required (Tenenbaum et al., 2011). As Valadao et al. (2012) demonstrate, keeping in mind information that may be relevant for detecting changes in environmental statistics can affect the ability to learn the statistical distribution that gave rise to that data. An impairment in patients’ abilities to integrate information, or to keep it in mind, will impair their ability to learn statistical regularities and affect their ability to create mental models of the environment. This will impact everything from adapting to new surroundings to benefiting from rehabilitation programs.

These ideas have guided our investigations of neglect patients’ ability to learn and update mental models. One of the first studies demonstrating that neglect patients have a representational impairment is the very elegant and famous study of Bisiach and Luzzatti (1978). Patients were asked to imagine how they would see a famous square in Milan. What the authors found is that patients could represent all the buildings presented on their imagined right, but failed to report those on their left. When the experimenters asked patients to imagine themselves standing on the opposite side of the square, so that the buildings they had previously neglected were now on their right, the patients reported those building but missed (i.e., neglected) those they had previously reported. Bisiach and Luzzatti (1978) concluded that patients demonstrate neglect even for their mental representations.

Another demonstration of a representational impairment in neglect comes from motor imagery. Danckert et al. (2002) have shown in one neglect patient, that imagining and creating mental representations of motor movements is impaired, while they do not show any impairment while actually *performing* those movements. In their study, the researchers asked one neglect patient to imagine a motor action, such as pointing toward targets of different sizes. The patient demonstrated normal movements, that conformed to expected speed-accuracy trade-offs (i.e., movement duration decreased with increasing target size), whereas imagined movements did not show such a pattern. That is, contrary to the actual movement, where the patient was faster to point to larger targets – which corresponds to the performance of healthy participants – when asked to imagine a movement for a given target, the patient did not show a relation between the time to imagine the movement and the size of the presented target, further demonstrating the challenge neglect patients have in creating accurate mental models – in this instance a model of an intended action (Danckert et al., 2002).

Similarly, other studies have shown impairments of updating using the double step saccade task (Duhamel et al., 1992). In this task, participants saccade to two successive targets that are extinguished in under 200 ms (i.e., prior to initiation of the first saccade). In order to accurately acquire the second target, an individual must anticipate the sensory consequences of the first saccade to update a mental representation of space. Results showed that a neglect patient was unable to accurately saccade to the second target when the first target was presented in contralesional space and the second target appeared in ipsilesional space, demonstrating an impairment in updating a mental representation of intended eye movements in space (Duhamel et al., 1992; see also Heide et al., 1995, 2001).

Many studies investigating which brain regions are involved in updating, decision-making, statistical learning, and novelty detection have found sets of structures that overlap those often injured in neglect. For example, the right hemisphere generally appears critical for priming and statistical learning (Kristjánsson et al., 2007; Turk-Browne et al., 2009). Roser et al. (2011) presented sequences of shapes with varying transitional probabilities in the left or right visual field of a split-brain patient. The patient could learn the statistical relationship of the shapes when they were presented to his left visual field, but not when they were presented on his right. The authors concluded that the right hemisphere plays an important role in statistical learning (Roser et al., 2011). Finally, the temporo-parietal junction (TPJ), a region commonly involved in neglect, has been identified in several studies as being important for representational updating (Clark et al., 2000; Downar et al., 2002; Mort et al., 2003; for a review, see Corbetta and Shulman, 2002; Husain and Rorden, 2003). In a study where changes in event related potentials (ERP) were studied based on novel or unusual events, it has been shown that the P300 component, localized to the TPJ, is increased in amplitude for novel events (Dien et al., 2003). The authors found that when information coming from the environment required an update of existing mental models, the electroencephalographic activity at the TPJ increased. The TPJ is also believed to be activated when attention needs to be directed toward behaviorally relevant events (Corbetta and Shulman, 2002). In their review, the authors suggest that the TPJ acts as a “circuit breaker,” important for redirecting attention toward salient information in the environment. Therefore, we hypothesize that TPJ might help to orient attention toward information that is useful to update mental models. Finally, other studies have identified the parietal cortex as being an important region involved in representational updating (Vuilleumier and Driver, 2007; Danckert et al., 2012a,b).

**Figure 5:**
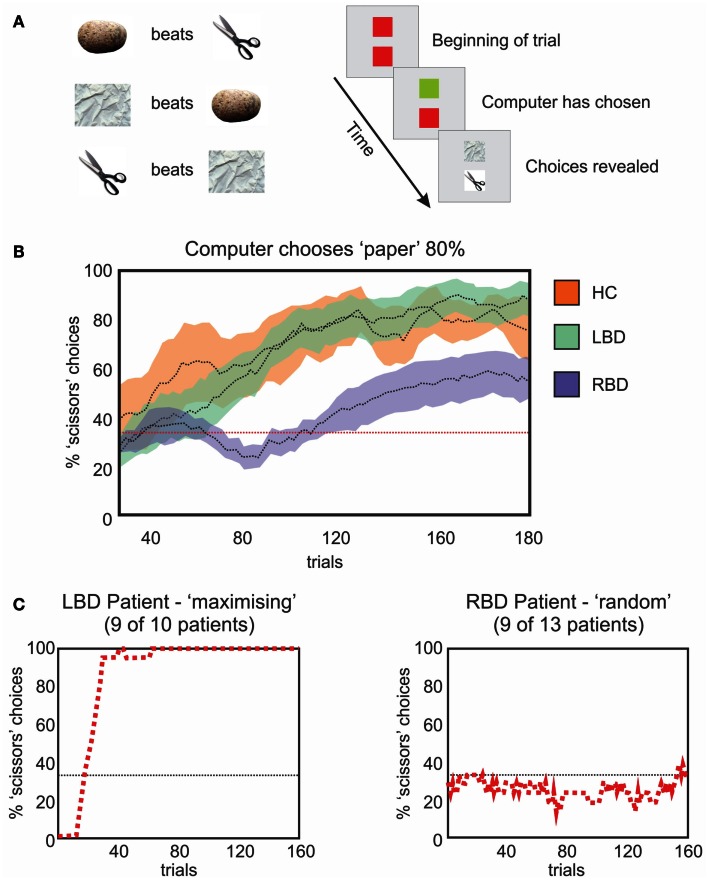
**(A)** Schematic representation of the rules that govern the RPS task (left) and a single trial (right). The upper square represents the computer’s choice, the lower square the participant’s choice. The computer’s square changes to green when a choice is made and the participant then make a choice, after which, both plays are revealed to indicate the result. **(B)** Moving average (20 trials) of optimal choices vs. the strong bias of the computer (i.e., 80% paper). HCs (orange) and LBD patients (green) exploit the bias. RBD patients fail to exploit the bias as efficiently. **(C)** Representative performances from a LBD (left) and RBD (right) patient. The LBD patient maximizes choosing the optimal play 100% of the time. The RBD patient continues to play randomly and uniformly.

We designed a study to investigate the ability of neglect patients to learn statistical distributions and to use the incoming information for creating and updating mental models (Figure 5; Danckert et al., 2012a). We had patients play the children game rock-paper-scissors against a computer opponent that covertly varied its play strategy. In the first block of trials, the computer opponent chose uniformly from the three options rock, paper, and scissors, and did so independent of the participant’s choice on any prior trials. The computer subsequently chose one item 80% of the time. Right brain damaged patients were not able to adapt their play to the heavily biased strategy of the computer, whereas control participants and left brain damaged patients did so without difficulty (Figure 5). We conclude that patients with right hemisphere injury (many of whom also had neglect) have difficulty using sequentially collected information from the environment to create mental models, to use such models to guide behavior, and to detect when the models need to be updated secondary to environmental changes. This type of impairment could easily depend on the deficits that we and others have observed in priming, temporal processing, statistical learning, and working memory capacity.

**Figure 6:**
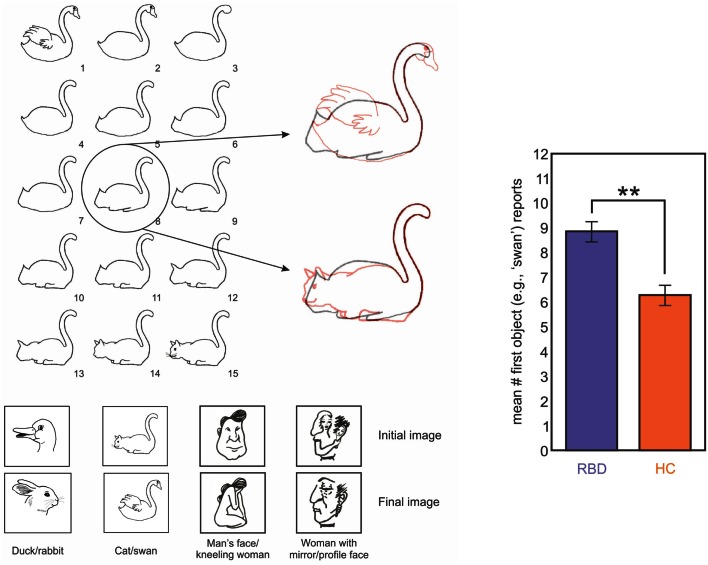
**Left panel: images used in one trial of the ambiguous figures task**. In this example a swan morphs into a cat. The middle image (#8) is highlighted overlaid on the first (swan) and last (cat) images in red to highlight the ambiguous interpretation for the middle image. The four image sets used are indicated below. Right panel: data from RBD (purple) and HC (orange) participants showing mean report of the first object (i.e., how long does the first perceptual model persist before participants switch to the second?). RBD patients reported the first object for significantly more trials than did HCs (Stoettinger et al., under review).

In order to evaluate impairments in mental model updating, and to do so in a way that was less dependent on statistical estimation, we tested the ability of right brain damaged patients to update mental representations of ambiguous figures (Figure 6; Christman et al., 2009; Stoettinger et al., 2013). We tested 12 patients (main age = 64, SD = 9): four had lesions of the parietal lobe and eight had lesions of the fronto-parietal area. A sequence of pictures began with a totally unambiguous representation of a common object (e.g., swan) and then gradually progressed through successive images that were slightly altered each time to eventually show a completely different, unambiguous item (e.g., cat; Figure 6). We used the number of stages for which patients retained their initial report of the original unambiguous figure in the sequence as a measure of updating. That is, when a person changed from reporting that they saw a swan to reporting a cat, they can be said to have updated their representation of the ambiguous figure. Results showed that right brain damaged patients persisted for longer than did controls in responding with the initial representation (e.g., swan) before adapting their responses to the figural changes (e.g., cat; Figure 6). Importantly, all subjects correctly identified the beginning and ending pictures, as well as catch trials in which simple geometric figures were inserted into the sequence. These data are in good agreement with those of Vocat et al. (2012), who tested right brain damaged patients with anosognosia on a riddle test. Participants listened to five increasingly specific clues (for example, for the targeted word “*airplane*,” they were given the clues: “*I have wings*,” “*I can fly*,” and then the last clue was “*I have wheels*”). The authors found that anosognosic patients reported higher levels of certainty regarding their initial guesses associated with the first clue (even those that were not particularly informative) and to preserve their response, although the next clues disconfirmed their guess. For example, with the clue “*my weight is approximately 300 grams*” and the target word “*heart*,” a patient guessed the word “*bread*,” and then with the next clue, “*I produce a regular sound*,” he persisted with the answer “*bread*” but justified it by saying it’s the noise that the knife makes when we cut bread (Vocat et al., 2012). The authors concluded that patients were impaired in creating and adapting beliefs to new information: they were overconfident about their initial guesses and failed to revise those guesses when successive clues were incongruent with that guess. Data from our studies on rock, paper, scissors (Danckert et al., 2012a), the ambiguous figures task (Stoettinger et al., 2013) and Vocat’s et al. (2012) riddle task are all consistent with the hypothesis that right brain damaged patients have difficulties in creating and updating mental models of the environment (Danckert et al., 2012a,b). Critically, these difficulties cannot be explained by recourse to deficits in spatial attention. So while previous rehabilitation attempts may succeed to some degree in improving deficits of spatial attention (Striemer and Danckert, 2007, 2010), they are unlikely to improve the more generic deficit in building accurate mental models and updating those models as environmental changes dictate.

## Implications for Rehabilitation Strategies

While neglect patients have trouble creating and updating mental models (Danckert et al., 2012a,b), this difficulty is not absolute. As the aforementioned studies of ambiguous figures (Stoettinger et al., 2013) and riddle tasks (Vocat et al., 2012) have shown, patients eventually get the correct answers; it just takes them longer to get there. The patients’ need more information and longer periods of time compared to healthy controls and this is where the rehabilitation strategies should focus.

**Figure 7:**
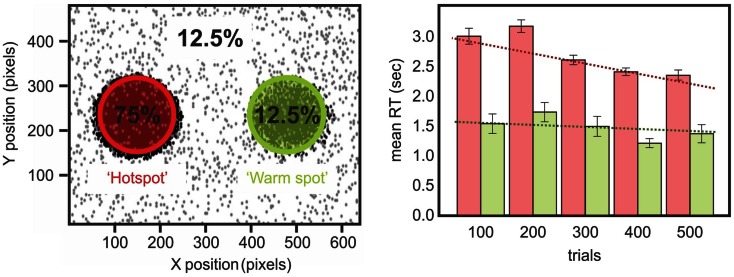
**Left panels show the distribution of the biased target positions while a chronic neglect patient performed a color discrimination task (Shaqiri and Anderson, 2012a, see also Figure 1)**. Targets were biased to appear 75% on a hotspot on the patient’s contralesional side and 12.5% on a mirrored region on his ipsilesional side called the warm spot. Right panels show the chronic neglect patient’s RT for the hot spot and the warm spot. There is a difference on the RT for the left and right-sided targets over the sessions. The patient improved his RT over the sessions for the targets presented on the hotspot, which was not the case for the targets presented on the warm spot.

If statistical learning is inefficient in neglect then maybe massing trials would be another approach for training a corrective bias in patients’ attention. This might make for an appropriate rehabilitation tool. To test this idea, we trained a chronic neglect patient by testing him over three different days on the paradigm of statistical learning adapted from Druker and Anderson (2010) (Figures 1 and 7). We analyzed whether the patient showed greater improvement in reaction time over trials for targets presented on the left compared to those on the right and found that after training, the patient was able to improve performance for the contralesional high probability region and become faster for targets in left, previously neglected space, although his performance did not reach the same speed as his RTs for right-sided targets (Figure 7). These results demonstrate that while patients with neglect have difficulties benefiting from the statistical distribution on their contralesional side, if they are given enough time to detect the targets (Kristjánsson et al., 2005) or if they are submitted to the regularities of the target position for a longer period of time (Figure 7), then they might benefit from the statistical regularities and improve their performance. Therefore, our data is in agreement with the studies of Geng and Behrmann (2002, 2006): although their protocol had a reduced number of positions and could have suffered from the confound of position priming, their patients with neglect were sensitive to the probability of the stimulus location, and this acted as a cue for directing attention. We demonstrated (Shaqiri and Anderson, 2012a,b, under review) that neglect patients have a preserved but attenuated priming effect, but are also sensitive to some extent to probability distributions, although a longer exposure duration is needed to demonstrate this sensitivity.

Taken together, these data could have important implications for the rehabilitation of neglect patients. First, the non-spatial features of neglect must be understood to be important contributors to the nature and recalcitrance of the clinical symptoms. Second, deficits in domains such as priming, temporal processing, and working memory may underlie deficits in mental model building and updating that can have pervasive effects on daily behavior and limit the benefits due to conventional rehabilitation. Our data also suggest that if given enough time and experience, neglect patients can benefit from regularities of their environment, as we have shown by training a neglect patient over three different days (Figure 7; Shaqiri and Anderson, 2012a). If considered when designing and testing rehabilitation techniques for neglect, the observations suggest new domains for intervention and emphasize that constant, regular biases with training over multiple sessions may help patients to develop the intrinsic biases that will improve performance across multiple tasks, and in activities of daily life. A rehabilitation approach that could exploit these data is virtual reality (VR). VR permits the flexible modulation of stimulus timing, exposure duration, and environmental regularities. This technique also permits creating personalized environments that match individual patients’ impairments. VR approaches to rehabilitation have already shown some promise for neglect patients (for a review, see Rose et al., 2005; Tsirlin et al., 2009), where, for example, VR rehabilitation has been used for training how to cross the street safely (Weiss et al., 2003; Katz et al., 2005).

## Conclusion

In the present review paper, we have presented different studies that demonstrate that beyond the spatial aspect of neglect, the disorder is linked with a range of other deficits, including working memory, temporal processing, motor imagery, statistical learning, and priming impairments. Taken together, this range of impairments make it extremely difficult for neglect patients to build accurate mental models of the environment and to update those models when contingencies change. In essence, this makes neglect a disorder of representational updating: a difficulty in using incoming information from the environment in order to create and then update mental models about that environment. It is a difficulty that most rehabilitation techniques available have not succeeded in overcoming. We have demonstrated that with enough time and information, some neglect patients can be trained to be sensitive to the statistical distribution and regularities from their environment and use that information to their benefit. As such, this may be a fruitful avenue for developing novel rehabilitative techniques for what has proven to be an extremely difficult disorder to treat.

## Conflict of Interest Statement

The authors declare that the research was conducted in the absence of any commercial or financial relationships that could be construed as a potential conflict of interest.
